# A Review of Turkish Self-Reporting Instruments for Sleep Problems in Children, Adolescents, and Adults

**DOI:** 10.5152/eurasianjmed.2023.23309

**Published:** 2023-12-01

**Authors:** Abdullah Bozkurt, Ömer Faruk Uygur, Esen Yıldırım Demirdöğen, Mehmet Akif Akıncı

**Affiliations:** 1 Department of Child and Adolescent Psychiatry, Atatürk University, Erzurum, Türkiye; 2 Department of Psychiatry, Atatürk University School of Medicine, Erzurum, Türkiye

**Keywords:** Adolescents, adults, children, self-reporting instruments, sleep scale

## Abstract

Many self-reported sleep instruments have been developed and adapted into many languages, including Turkish, and their psychometric properties have been examined. This study aims to present a literature review on self-report instruments adapted into Turkish and used to assess different aspects of sleep in child, adolescent, and adult populations. Terms related to sleep self-report instruments were applied to 4 search engines: PubMed, EMBASE, Scopus, and Google Scholar. The search engines were searched for articles published until October 2023. Assessment instruments whose psychometric properties were not evaluated in Turkish language were excluded from the study. This study defined 27 sleep instruments, including 9 child and adolescent and 18 adult sleep instruments. Increasing the availability of Turkish sleep tools for children and adult will improve the chance of intervention by increasing the identification of sleep disorders.

## Introduction

Sleep is a temporary, periodic, and psychophysiological process characterized by decreased awareness, sensory functions, and voluntary muscle movements.^1^ Sleep is an important physiological need to maintain the functioning of the human brain.^2,3^ Sleep takes up approximately one-third of a human life.^4^ A restorative and optimal sleep is crucial for promoting overall well-being and maintaining a positive state of health and contentment.^5^

Research reports that sleep problems are prevalent in children, adolescents, and adults. Insomnia is also the most common symptom of sleep problems in the population. The most popular model for insomnia is Spielman’s 3P model. According to this three-factor model, acute insomnia begins when stressful life events are added to predisposing factors, such as a more anxious personality. Negative cognitions about sleep quality and insomnia, such as worry, rumination, and catastrophic thoughts, also cause chronic insomnia.^6^ In addition, maladaptive behaviors that do not comply with sleep hygiene, such as staying in bed for a long time and making too much sleep effort, contribute to the chronicity of insomnia.^7^ Insomnia is related to an increased risk of psychiatric problems such as attention and behavioral problems, impulsivity, impaired social functioning, and digital addiction.^8-10^ There are some objective methods for evaluating insomnia. Polysomnography and wrist actigraphy are the main objective methods, but they are time-consuming and require equipment. On the other hand, multiple psychometric sleep instruments are suitable for practical use.^11^ Self-reported sleep instruments are the preferred method for evaluating insomnia in children and adults, given the limited availability and high costs of objective testing devices. Many self-reported sleep instruments have been developed and adapted to many languages, including Turkish, and their psychometric properties have been examined. The sleep instruments might be grouped into 3 categories in the literature:

1. Instruments assessing insomnia symptoms and general sleep problems.
2. Instruments assessing the cognitive aspect of insomnia.
3. Other self-reported tools (about circadian rhythm, chronotype, etc.).

Numerous systematic evaluations have been conducted on self-assessed sleep instruments. However, new scales are constantly added to the existing ones, or studies are carried out on adapting old scales to different languages and examining their psychometric properties. Our study aimed to review and update the literature on self-reported sleep assessment tools in Turkish for pediatric, adolescent, and adult populations.

### Materials and Methods

The search strategy utilized the following combined search terms: “sleep” and (“infant*” or “child*” or “adolescent*” or “adult*”) and (“questionnaire*” or “instrument*” or “scale*” or “checklist*” or “assessment*” or “diary*” or “record*” or “report*” or “test*” or “measure*”) and (“turkey*” or “turkish*”). These search terms were applied to 4 search engines: PubMed, EMBASE, Scopus (including web crawling), and Google Scholar (also including web crawling). The search engines searched for articles published until October 2023. Assessment instruments whose psychometric properties were not evaluated in Turkish were excluded from the study.

## Results

Some sleep instruments could not be accessed because of non-response, outdated contact information, and copyright issues, and as a result, a pool of 27 instruments was obtained. In Turkish, 18 sleep instruments were for adults and 9 for children and adolescents.

## Instruments Assessing Insomnia Symptoms and General Sleep Problems

### Instruments for Adults

#### Pittsburgh Sleep Quality Index

The Pittsburgh Sleep Quality Index (PSQI) is one of the most commonly used scales in sleep research. It is a self-report questionnaire that assesses sleep quality and sleeping habits within the last month. The PSQI consists of 24 questions, and 5 of them are answered by a sleep partner. Nineteen items evaluate 7 components: subjective sleep quality, sleep latency, sleep duration, habitual sleep efficiency, sleep disturbances, use of sleep medication, and daytime dysfunction. All items are scored between 0 and 3. The partner’s answers are not included in the total score. The scores of 7 components consisting of 19 questions are scored with a specific score model, and the total score is obtained after the scores of 7 components are scored according to this model. Individuals with a total score above 5 are categorized as poor sleepers. The PSQI was developed by Buysse et al^12^ in 1988. Agargün et al^13^ adapted PSQI to the Turkish language and conducted validity–reliability analyses in 1996. The Turkish PSQI has a single-factor structure similar to the original scale and solid psychometric properties.^12^

#### Insomnia Severity Index

Bastien et al^14^ developed the Insomnia Severity Index (ISI) to assess the severity of insomnia. The ISI comprises a set of 7 items designed to assess the following factors: difficulty initiating sleep, earlier awakening, contentment with present sleep patterns, disruption of daily activities, perceptibility of impairments associated with sleep difficulties, and distress resulting from sleep difficulties. The ISI items are each assigned a score ranging from 0 to 4, and the cumulative score spans from 0 to 28. A higher score indicates a more severe case of insomnia. Four categories comprise the total score: 0-7 indicates no clinically significant insomnia; 8-14 indicates subthreshold insomnia; 15-21 indicates moderate insomnia; and 22-28 indicates severe insomnia. A total score of 15 or more indicates clinical insomnia, while a score of 8 or less may indicate remission following treatment for insomnia. The ISI, adapted to Turkish by Boysan et al^15^ in 2010, had strong psychometric properties with a 2-factor structure (daily functionality and sleep quality).

#### Bergen Insomnia Scale

Pallesen et al^16^ developed the Bergen Insomnia Scale (BIS) in 2008. They prepared BIS according to *Diagnostic and Statistical Manual of Mental Disorders* (Fourth Edition, Text Revision) (American Psychiatric Association, 2000) insomnia diagnostic criteria. It has 6 items and questions about insomnia in the past month. The first 4 items relate to sleep onset, maintenance, early morning waking, and nonrestorative sleep. The last 2 items assessed sleep satisfaction and daytime impairment. Each item is scored with scores ranging from 0 to 7 days; the total score varies between 0 and 42. The original scale study was conducted in the general population, student groups, and patient groups. While the scale showed a 2-factor structure in the student and patient samples, it showed a single-factor structure in the general sample. Its internal consistency was sufficient (Cronbach’s alpha = 0.79). The scale was adapted to Turkish by Bay and Ergun in 2018.^17^ The Turkish BIS, with 6 items and 2 factors, has demonstrated adequate psychometric properties (Cronbach’s alpha = 0.72, nocturnal symptoms (items 1, 2, and 3), daytime symptoms (items 4, 5, and 6).^17^

#### General Sleep Disturbance Scale

The General Sleep Disturbance Scale (GSDS) is a 21-item self-assessment tool that evaluates sleep disorders. It questions how frequently specific experiences occurred over the past week in 6 episodes. Difficulties initiating sleep (1 item), awakening during sleep (2 items), sleep quality (3 items), sleep duration (2 items), diurnal lethargy (7 items), and the use of substances to induce sleep (6 items) comprise the 6 sections. Answers range from 0 (no days) to 7 (every day) for each question. The total score varies between 0 and 147. Responses of 3 days or more to each item correspond to the diagnostic criteria for insomnia in the *Diagnostic and Statistical Manual of Mental Disorders* (Fifth Edition). The GSDS was developed by Lee^18^ in 1992, and Cronbach’s alpha coefficient was 0.88. Senlikci et al^19^ adapted GSDS to the Turkish language and calculated psychometric properties in stroke patients. Turkish GSDS has good internal consistency (Cronbach’s alpha = 0.85).

#### Jenkins Sleep Scale

The Jenkins Sleep Scale (JSS) is a 4-item self-report instrument to assess sleep disturbances experienced over the past month. It includes queries on challenges in initiating sleep, nocturnal awakenings, difficulties in maintaining sleep, and experiences of nonrestorative sleep. Participants are required to indicate the frequency of these issues, denoting the number of days per month each problem occurs.^20^ Each item is rated on a 6-point Likert scale (note at all = 0, 1-3 days = 1, 4-7 days = 2, 8-14 days = 3, 15-21 days = 4, and 22-28 days = 5). The total score ranges from 0 to 20. Eleven points is the cutoff point, and a score >11 is shown as a high frequency of sleep disturbances. The JSS has been adapted to many languages and tested psychometric properties in clinical populations such as rheumatoid arthritis, psoriatic arthritis, ankylosing spondylitis, fibromyalgia, and epilepsy.^20^ Ulutatar and Unubol^21^ adapted JSS to the Turkish language in fibromyalgia patients and showed adequate psychometric properties (Cronbach α = 0.741).

#### SCOPA Sleep Scale

SCOPA Sleep Scale was developed in 2003 by Marinus et al.^22^ It consists of 12 items in total. The assessment includes 5 items dedicated to evaluating nighttime sleep, 6 items for appraising daytime sleepiness, and a single item for assessing overall sleep quality. Scores can reach up to 15 for nighttime sleep and 18 for daytime sleepiness. Elevated scores in both of these subdimensions indicate a greater prevalence of sleep disturbances. The threshold for categorizing sleep quality as poor is set above 4 for the daytime sleepiness subscale and above 6 for the nighttime sleep subscale.^22^ The Turkish adaptation was conducted by Sönmez Kongur et al^23^ in 2022. The scale showed a 2-factor structure and strong psychometric properties, similar to the original scale (Cronbach’s α = 0.907 for the nighttime sleep subdimension and 0.906 for the daytime sleepiness subdimension).

#### The Postpartum Sleep Quality Scale

The Postpartum Sleep Quality Scale (PSQS) is a scale developed for use in postpartum period sleep problems. This scale has 2 factors and contains 14 items. Each item has a 5-point Likert structure; the total score varies between 0 and 56 points. There is no cutoff value, and high scores are associated with poor sleep quality. The original scale has a Cronbach alpha value of 0.81. It was adapted into Turkish by Boz and Selvi in 2017.^24^ Turkish PSQS has a 3-factor structure and a high internal consistency coefficient (0.92) (Factor 1: infant night care-related daytime dysfunction; Factor 2: physical symptoms-related sleep inefficiency; Factor 3: sleep quality or sleep efficiency).^24^

#### Richards–Campbell Sleep Questionnaire

The Richard–Campbell Sleep Questionnaire (RCSQ) is a 6-item scale that assesses the depth of nighttime sleep, time to fall asleep, frequency of awakening, duration of wakefulness, quality of sleep, and ambient noise level. Each item on the scale is assessed using a visual analog scale technique, with scores ranging from 0 to 100. A score within the “0-25” range signifies a very poor quality of sleep, whereas a score in the “76-100” bracket indicates very good sleep. The psychometric properties of the Turkish version of the RCSQ were found to be good and acceptable.^25^

#### Medical Outcomes Study Sleep Scale

The Medical Outcomes Study (MOS) sleep scale, which comprises 12 items, assesses individuals’ subjective sleep experiences in a variety of categories. The scale is a generic assessment tool used to evaluate sleep outcomes, relying on self-reported data provided by patients. The participants are instructed to recollect the preceding 4-week period and respond to inquiries pertaining to that time period.^26^ Ten of the 12 questions demand 6-point Likert-type responses, while one requires 5-point responses. Akcay et al^27^ performed Turkish adaptation and validation of the scale in patients with obstructive sleep apnea.

### Instruments for Children and Adolescents

#### Sleep Disturbances Scale for Children

This scale was developed by Bruni et al^28^ to evaluate sleep-related problems and specific sleep disorders that may be observed in children and adolescents aged 6-16 years. The Sleep Disturbances Scale for Children (SDSC) is expected to be evaluated by the caregivers of children and adolescents, considering the last 6-month period. There are 26 items on the scale, and a Likert-type evaluation system graded between 1 and 5 points is used for each item. The first question asks for the total amount of sleep covering the whole night, and the second asks for sleep onset time. In items 3-26 following these questions, the frequency of occurrence of the stated condition is asked to be marked. The total score that can be obtained from SDSC may vary between 26 and 130, and the cutoff score was found to be 39. Sleep disorders observed in this age group are divided into 6 subcategories in the SDSC: problems related to the transition to and maintenance of sleep, problems related to sleep termination (waking up), respiratory problems observed during sleep, problems related to high levels of sleepiness, problems related to the transition between sleep and wakefulness, and excessive sweating during sleep. The Turkish validity and reliability study of this scale was conducted by Ağadayı et al.^29^

#### Children’s Sleep Habits Questionnaire

It is a frequently used scale in the evaluation of sleep-related problems that may occur in childhood and the sleep habits of children. The original version of the Children’s Sleep Habits Questionnaire (CSHQ) was developed by Owens et al.^30^ The questionnaire consists of 45 items, 33 scored items, and 7 open-ended items. The CSHQ has 8 subheadings: resistance to going to bed, prolonged sleep transition time, sleep-related anxiety, the total amount of sleep, respiratory distress during sleep, parasomnia-related conditions, sleep disturbances during the night, and sleepiness during the day. Scored items are assessed on a 3-point Likert-type scale. The CSHQ was completed by the mother and/or father retrospectively over the previous week. The higher the overall score on the scale, the greater the sleep problems. Scores above 41 indicate a medically significant deterioration. The Turkish validity and reliability study of the questionnaire was performed by Perdahlı Fiş et al.^31^

#### Pediatric Daytime Sleepiness Scale

The scale used in evaluating sleepiness and treatment responses in studies on sleep disorders was adapted for children and adolescents by Drake et al.^32^ The Pediatric Daytime Sleepiness Scale (PDSS) consists of 8 items graded on a 4-point Likert scale.^32^ Total scores on the scale vary between 0 and 32, with higher scores indicating more intense daytime sleepiness. The assessment of the scale’s validity and reliability in Turkey was carried out by Bektaş et al.^33^

### *Diagnostic and Statistical Manual of Mental Disorders* (Fifth Edition) Level 2 Sleep Disorders Scale

It is a tool designed to be used in the initial evaluation process and the follow-up of children and adolescents with sleep problems. It consists of a total of 8 items. The scale provides a 5-point Likert-type rating. The total score ranges from 8 to 40. Higher scores indicate more severe symptoms associated with sleep disturbance. Özerk Erkuran et al^34^ found the reliability and validity of the Turkish version to be adequate.

### Pediatric Sleep Questionnaire

It was developed by Chervin et al^35^ to investigate respiratory problems experienced during sleep.^35^ The Pediatric Sleep Questionnaire is categorized under 3 main headings: activities observed during the night and at bedtime, activities and problems observed during the daytime, attention problems, and hyperactivity findings. Öner et al^36^ found the PSQ valid and reliable in Turkish.

### The Adolescent Sleep–Wake Scale

The Adolescent Sleep–Wake Scale (ASWS) assesses sleep quality in adolescents aged 12-18 using a 28-item tool.^37^ Essner et al^38^ developed a 10-item short form of the ASWS. Better sleep quality is indicated by higher scores. The short form has 3 subscales: falling asleep and reinitiating sleep, returning to wakefulness, and going to bed. The Turkish ASWS was found by Bozkurt et al^39^ to be a valid and reliable instrument.

## Instruments Assessing the Cognitive Aspect of Insomnia

## Instruments for Adults

### Dysfunctional Beliefs and Attitudes About Sleep Scale

The Dysfunctional Beliefs and Attitudes About Sleep Scale (DBAS) is a self-report scale that contains 16 dysfunctional sleep-related items. It has a 10-point Likert structure, with higher scores reflecting more dysfunctional beliefs about sleep.^40^ It was determined that the Turkish-adapted DBAS showed good psychometric properties (Cronbach α = 0.82, test-retest stability *r* = 0.83).^41^

#### Anxiety and Preoccupation about Sleep Questionnaire

The Anxiety and Preoccupation about Sleep Questionnaire (APSQ) is a 10-point Likert scale consisting of 10 questions. The scale has 2 subscales: the first is about anxieties about the outcomes of insufficient sleep, and the second is about worries about the uncontrollability of sleep. Tang and Harvey developed the scale in 2004 to measure sleep-related anxiety.^42^ Higher scores represent more significant insomnia-related anxiety. In 2022, Uygur et al^42^ adapted APSQ to the Turkish language. The Turkish APSSQ has robust psychometric properties and an excellent internal consistency coefficient (0.95).^42^

#### Insomnia Catastrophizing Scale

The Insomnia Catastrophizing Scale (ICS) was developed to evaluate catastrophic thoughts related to the nighttime symptoms of insomnia and the daytime dysfunction it causes.^43^ It consists of a total of 17 items and 2 subscales. The first part, which consists of 11 items, evaluates the catastrophic thoughts that occur during the night, ICS-night, and the second part, which consists of 6 items, considers the catastrophic ideas that occur during the day. The answers were designed as 6-Likert answers (0 = never, 5 = always). The psychometric properties of the original scale developed were examined in a large sample.^43^ In the studies, the factor structure of both subscales was examined as 2 different scales. Five items were excluded from the final version of the Turkish ICS, and it showed strong psychometric properties.^43^

#### Glasgow Sleep Effort Scale

The Glasgow Sleep Effort Scale (GSES) consists of 7 items and a 3-point Likert structure. It measures cognitive (e.g., “I must sleep” schema) and behavioral (e.g., performance effort) sleep effort. A high total score means a high sleep effort. It was found that sleep effort was positively correlated with insomnia severity and dysfunctional beliefs and attitudes about sleep.^44^ Turkish adaptation was conducted, and Turkish GSES showed good psychometric properties. Clinical insomnia required a GSES score of at least 6 points, while poor sleepers needed at least 3 points.^45^

## Other Self-Reported Tools (About Circadian Rhythm, Chronotype, Sleep Hygiene, Sleep Reactivity)

### Instruments for Adults

#### Sleep Hygiene Index

The Sleep Hygiene Index (SHI) is a 13-item self-reported scale that evaluates environmental and behavioral variables that lead to poor sleep. The total score on the scale varies between 13 and 65 points. Higher scores are associated with worse sleep hygiene. The SHI was adapted into Turkish by Özdemir et al^46^ Turkish SHI exhibited a single-factor structure with a sufficient internal consistency coefficient (Cronbach alpha values of 0.70 and 0.71 for the community sample and patients with major depression, respectively).

#### Epworth Sleepiness Scale

The Epworth Sleepiness Scale (ESS) is currently the most widely used method of assessing sleepiness in sleep disorders. Johns developed a simple, self-administered, English-language questionnaire. It asks individuals to rate their likelihood of falling asleep in 8 common situations in the past month using a scale from 0 to 3 (0 = no chance of dozing, 1 = slight chance of dozing, 2 = moderate risk of dozing, 3 = high chance of sleeping). The ESS score, which ranges from 0 to 24, is the total of the 8 item scores. Higher ESS scores imply more sleepiness during the day. Izci and colleagues^47^ translated and validated it into Turkish.

#### Morningness–Eveningness Questionnaire

The Morningness–Eveningness Questionnaire (MEQ) is a 19-item scale developed to evaluate a person’s chronotype. The scale assesses the individual’s physical and psychological performance and favorite periods for various activities. Scores range from 16 to 86, with scores between 16 and 30 indicating definitive and 70-86 indicating definitive morning type. It was developed by Horne and Östberg in 1976. The Turkish version of the MEQ was developed and verified by Pündük et al^48^ (2005). The researchers obtained a test-retest correlation value of 0.84.

#### Munich Chronotype Questionnaire

The Munich Chronotype Questionnaire (MCTQ), developed by Roenneberg et al^49^, is a self-rated scale consisting of 17 items that calculate chronotype by determining the midpoint between sleep onset and offset on nonworking days. According to MEQ, the calculation is a little more complicated. It was adapted into Turkish by Erdoğan et al who reported that the test-retest reliability coefficient of the MCTQ-TR was calculated as *r *= 0.643 (*P *< .05).

#### Ford Insomnia Response to Stress Test

The Ford Insomnia Response to Stress Test (FIRST) is a self-report instrument utilized to evaluate sleep reactivity. Sleep reactivity refers to the potential occurrence of insomnia as a result of particular stress-inducing circumstances. Each item is rated: not likely = 1, somewhat likely = 2, moderately likely = 3, and very likely = 4. A total score between 9 and 36 points can be yielded from the scale. Elevated scores indicate increased degrees of sleep reactivity. The scale has been adapted to Turkish.^50^

#### Children’s Chronotype Questionnaire

It was developed based on the MCTQ to evaluate circadian rhythm characteristics in childhood.^51^ The Children’s Chronotype Questionnaire (CCQ) consists of a total of 16 items. Those who score ≤23 points are defined as “morning type,” those who score between 24 and 32 points are defined as “intermediate type,” and those who score ≥33 points are defined as “evening type.” Dursun et al^52^ adapted the CCQ into Turkish.

## Instruments for Children and Adolescents

### Morningness–Eveningness Scale for Children

The Morningness–Eveningness Scale consists of 10 self-reported questions with a Likert-type response format. The scale score ranges from 10 to 43. A score between 10 and 21 indicates an “evening type,” between 22 and 34 an “intermediate type,” and between 35 and 43 a “morning type.” The validity and reliability of the scale have been established in Turkish.^53^

### Infant Sleep Scale for Parents

The scale consists of 11 items and 3 subdimensions: “sleep routines,” “sleep autonomy,” and “screen media in sleep environment.”^54^ The content validity index value of the scale items is 0.97. The total Cronbach’s alpha coefficient of the scale was 0.73. The ISSP can be used as a valid and reliable measurement tool in Turkish.^55^

## Discussion

This study defined 27 sleep instruments, including 9 child and adolescent and 18 adult sleep instruments.

The utilization of self-report scales holds significant importance in identifying and surveilling sleep disorders among adult individuals. Based on our extensive research, it has been shown that a multitude of scales exist in the Turkish context that pertain to the assessment of insomnia and sleep quality among adult individuals. Nevertheless, it should be noted that the existing adult scales in the Turkish language have limitations when it comes to assessing sleep disorders such as idiopathic hypersomnia, narcolepsy, and parasomnias.

There is a prevalent lack of awareness that sleep issues in children are relatively common. These problems can be easily mistaken for other pediatric conditions, lowering the possibility of appropriate treatment. Conducting a systematic assessment for sleep problems can help identify academic, behavioral, health, and quality of life issues early on.^56^ Our review revealed a lack of Turkish tools to assess sleep quality, sleep hygiene, and cognitive aspects of sleep disorders in adolescents and children. Sleep hygiene screening instruments can improve sleep efficiency by identifying sleep-related misbehavior. Developing sleep scales completed by parents and children to obtain reliable information may also be helpful. Increasing the availability of Turkish sleep tools for children and adolescents will improve the chance of intervention by increasing the identification of sleep disorders.
